# Genotyping and drug resistance patterns of *M. tuberculosis *strains in Pakistan

**DOI:** 10.1186/1471-2334-8-171

**Published:** 2008-12-24

**Authors:** Mahnaz Tanveer, Zahra Hasan, Amna R Siddiqui, Asho Ali, Akbar Kanji, Solomon Ghebremicheal, Rumina Hasan

**Affiliations:** 1 Department of Pathology and Microbiology, The Aga Khan University, Stadium Road Karachi, Pakistan; 2 Department of Community Health Sciences, The Aga Khan University, Stadium Road Karachi, Pakistan; 3 Department of Bacteriology, Swedish Institute for Infectious Diseases Control, Stockholm, Sweden

## Abstract

**Background:**

The incidence of tuberculosis in Pakistan is 181/100,000 population. However, information about transmission and geographical prevalence of *Mycobacterium tuberculosis *strains and their evolutionary genetics as well as drug resistance remains limited. Our objective was to determine the clonal composition, evolutionary genetics and drug resistance of *M. tuberculosis *isolates from different regions of the country.

**Methods:**

*M. tuberculosis *strains isolated (2003–2005) from specimens submitted to the laboratory through collection units nationwide were included. Drug susceptibility was performed and strains were spoligotyped.

**Results:**

Of 926 *M. tuberculosis *strains studied, 721(78%) were grouped into 59 "shared types", while 205 (22%) were identified as "Orphan" spoligotypes. Amongst the predominant genotypes 61% were Central Asian strains (CAS ; including CAS1, CAS sub-families and Orphan Pak clusters), 4% East African-Indian (EAI), 3% Beijing, 2% poorly defined TB strains (T), 2% Haarlem and LAM (0.2). Also TbD1 analysis (*M. tuberculosis *specific deletion 1) confirmed that CAS1 was of "modern" origin while EAI isolates belonged to "ancestral" strain types.

Prevalence of CAS1 clade was significantly higher in Punjab (P < 0.01, Pearsons Chi-square test) as compared with Sindh, North West Frontier Province and Balochistan provinces. Forty six percent of isolates were sensitive to five first line antibiotics tested, 45% were Rifampicin resistant, 50% isoniazid resistant. MDR was significantly associated with Beijing strains (P = 0.01, Pearsons Chi-square test) and EAI (P = 0.001, Pearsons Chi-square test), but not with CAS family.

**Conclusion:**

Our results show variation of prevalent *M. tuberculosis *strain with greater association of CAS1 with the Punjab province. The fact that the prevalent CAS genotype was not associated with drug resistance is encouraging. It further suggests a more effective treatment and control programme should be successful in reducing the tuberculosis burden in Pakistan.

## Background

Tuberculosis (TB) remains a major cause of morbidity and mortality worldwide, causing more than 2 million deaths a year [1,2]. Pakistan with a population of 140 million and a growth rate of 3.5% [3] has the seventh highest tuberculosis rate despite widespread BCG vaccination. TB prevalence in Pakistan can be attributed to poor health care systems and limited diagnostic and treatment modalities for TB[4]. The TB problem is also compounded by multi-drug resistance (MDR, resistance to at least rifampicin and isoniazid), WHO report suggests that globally 3% of *M. tuberculosis *isolates are MDR-TB[5]. Resistance to TB drugs is recognized in Pakistan[6,7]. While community based information is lacking, laboratory data suggests an increasing frequency of MDR from 14% in 1999 to 28% in 2004[6] and 47% in 2006[8].

International databases such as the SpolDB4.0 have revealed the clonal structure of *M. tuberculosis *isolates in different geographical settings. SpolDB4.0 data base further defines super families specific to certain locations[9]. Genotypic information has further expanded our understanding of strain prevalence and transmission [10-13]. A few predominant genotypes circulating throughout the world e.g. Beijing, Haarlem, and African clusters have been associated with a number of major outbreaks [14-16]. These major strain groups have been described as being predominant pathotypes in the world [17]. The abundance of polymorphism indicates that transposition and homologous recombination are the major events contributing to the diversity of *M. tuberculosis *strains [18]. In addition, polymorphism seen with different molecular markers also describes mutual association. This supports the hypothesis that *M. tuberculosis *has a strong clonal population structure [18]. In support of phylogeographical population structure of *M. tuberculosis*, differences in strain genetics may be responsible for the variation in BCG efficacy [19-22].

Predominant *M. tuberculosis *clades from the Indian sub-continent include Central Asian strain (CAS) [9,23] and Beijing strains [11,24-27]. Central Asian strain 1 (CAS1) are defined by absence of spacers 4–7 and 23–34 [28]. While, Beijing strains were characterized with the absence of 1–34 spacers in direct repeat region (DR). Beijing strains are reported to constitute about 50% of strains in far East-Asia and 13% of isolates globally[29]. East African-Indian strains, the T clade and Haarlem strains have also reported from India, Afghanistan and Iran[30,31]. In Pakistan predominance of CAS1 (39%) with a 6% prevalence of Beijing isolates has previously been reported [32].

Globally, MDR-TB outbreaks have been associated with Beijing and Haarlem families [33,34]. In order to understand the population structure of *M. tuberculosis *in Pakistan, strains from the four provinces, Punjab, Sindh, Balochistan and NWFP were spoligotyped. Genotypic information was correlated with drug resistance to determine association between strain types and MDR. Predominant clades obtained were further analyzed to distinguish between "ancestral" versus "modern" lineages of tubercle bacilli based on the presence or absence of the TbD1 region.

## Methods

### Mycobacterial strain collection

This study was conducted on *M. tuberculosis *strains isolated at the Aga Khan University Hospital (AKUH) in Karachi during the 3 year period 2003–2005. Specimens were from collection points situated in all four provinces of Pakistan. AKUH is a tertiary care hospital in Karachi and its clinical microbiology laboratory receives specimens through more than 100 collection points situated in all four provinces of Pakistan. All samples were delivered within a period of 24 hrs of collection and were processed for culture and sensitivity testing. Strains were stored at -70°c in 15% glycerol phosphate broth. During the study period 6,067 samples were processed from which 2208 strains were isolated. Based on the stratified random sampling method[32], a total of 926 strains were included in this study (It was ensured that only one sample per individual is included in our analysis). The largest 234 (25%) were from 14 different locations in Karachi. A further 691 strains were from the 4 provinces of Pakistan (excluding Karachi); 256 from Punjab, 224 from Sindh, 207 from the North West Frontier Province (NWFP) and 5 from Balochistan. We were not able to classify patients on basis of prior therapy since treatment history was not available. The *M. tuberculosis *isolates studied included both pulmonary (n = 850) and extra-pulmonary (n = 76) samples.

### Microbiological methods

#### Mycobacterial cultures and antibiotic susceptibility testing

Mycobacterial cultures were performed on liquid as well as solid media. Respiratory samples were decontaminated using N-acetyl-L-cysteine (NALC) sodium hydroxide prior to culture. Samples from sterile sites were processed without decontamination[35]. All specimens were concentrated by centrifugation (3000 × g) for 30 minutes and sediments cultured at 37°C using BACTEC 460 (Becton Dickinson Diagnostic Instruments Systems) and Lowenstein Jensen (LJ) medium. The growth index of inoculated BACTEC vials was checked for four weeks, LJ slants were incubated for up to 8 weeks. *M. tuberculosis *was identified by BACTEC NAP TB differentiation test (Becton Dickinson, USA).

Susceptibility testing was performed using standard agar proportion method on enriched Middle brook 7H10 medium (BBL) at the following final drug concentrations; rifampicin 1 ug/ml and 5 ug/ml, isoniazid 0.2 ug/ml and 1 ug/ml, streptomycin 2 ug/ml and 10 ug/ml and ethambutol 5 ug/ml and 10 ug/ml, ethionamide 5 ug/ml, capreomycin 10 ug/ml, cycloserine 30 ug/ml and ciprofloxacin 2 ug/ml. [36-38]. Pyrazinamide sensitivity was carried out using BACTEC 7H12 medium pH6.0 at 100 ug/ml (BACTEC™ PZA test medium, Becton Dickinson USA) in accordance with manufacturers instructions. To ensure selection of high level resistance strains for purposes of this study however, only resistance to the higher concentrations were used for analysis. Multidrug resistance (MDR) was defined as resistance to at least isoniazid and rifampicin.

#### Molecular methods

*Mycobacteria *were cultured on 7H10 Middle brook agar. DNA extraction was carried out from mycobacterial colonies using the CTAB method[39]. Spoligotyping was carried out using a commercially available kit from Isogen Bioscience BV, Maarssen, The Netherlands according to the manufacturer's instructions. Spoligotyping based on the 43 spacers of the DR region of *M. tuberculosis *complex was carried out using primers DRa 5'GGTTTTGGGTCTGACGAC3' and DRb 5'CCGAGAGGGGACGGAAAC 3' as originally described by Kamerbeek et al[24].

#### TbD1 analysis

TbD1; "*M. tuberculosis *specific deletion 1" consists of two genes encoding membrane protein (mmpS6 and mmpL6). TbD1 was originally identified as a 2153-bp fragment[40]. TbD1 deletion analysis was done by PCR as described by Brosch et al[41]. Two isolates from each cluster of CAS1 and its sub-families were tested for both primers complementary to flanking sequences and internal sequences in order to confirm deleted region (TbD1) in our Mycobacterium isolates. Sequences inside or flanking regions were obtained from the website [11].

### Data analysis

Spoligotyping results were entered in the Bionumerics Software, Applied Maths Program, BioSystematica, UK. Dendrograms were generated using the unweighted pair-group method with arithmetic averages (UPGMA) calculation. A cluster (shared types) was defined as two or more isolates from different patients with identical spoligotype patterns, whereas, non-clustered strains had 'orphan' spoligotype patterns. The spoligotypes were compared with the most prevalent *M. tuberculosis *subfamilies as identified by the World Spoligotyping Database SpolDB4.0 of Pasteur Institute of Guadeloupe [42]. The SpolDB4.0 information system is an automated Access-based labeling and matching system for spoligotyping. SpolDB4.0 which included ~40,000 isolates split into 1,939 shared types and ~3,530 orphan profiles. We compared each of our clusters with the shared types (STs) present in SpolDB4.0 [29].

Pearson's Chi-squared test was used to determine statistical association between strain types and specific parameters, Statistical Package for Social Science Software (SPSS, USA) was used for analysis. P values < 0.05 were considered significant. Considering that the isolates were independent the association between cluster types and drug resistance was analysed by the use of multilogistic regression model. The magnitude of the association was estimated by using the odds ratio (OR) and 95% confidence interval (CIs)

## Results

### Study population

A total of nine hundred and twenty six *M. tuberculosis *clinical isolates were studied. These were from pulmonary (n = 850) and extra-pulmonary (n = 76) sources. Of the pulmonary isolates studied, 411 (48%) were from the province of Sindh of which 195 (23%) were from Karachi; 232 (27%) were from the province of Punjab; 202(22%) from the North West Frontier Province (NWFP) and 5 (0.4%) from Balochistan. Out of seventy six extra-pulmonary isolates 47 (5%) were from Sindh, 24 (3%) from Punjab and 5 (0.5%) from NWFP.

An age-wise analysis of the *M. tuberculosis *clinical isolates showed that four hundred and forty seven (48%) isolates were from patients aged 15–30 years (214 males, 233 females), 246 isolates (26%) were from patient's aged 31–45 years (141 males, 105 females), while 233 isolates were from the 45+ years (25%) age group (154 males, 79 females).

### Spoligotyping of *M. tuberculosis *isolates

All *M. tuberculosis *strains were spoligotyped and their data analyzed using UPGMA calculation in the Bionumerics software. Seven hundred and twenty one (78%) isolates grouped into 59 different "shared types" while 205 (22%) isolates had 'orphan' spoligotypes. Central Asian strains 61% (n = 568) were found to be the most predominant genotype. Within the CAS genogroup, the CAS1 strain was predominant while, different levels of similarity were noted between CAS1 and other strains in the study population; 17 strains had 96% similarity, 22 strains had 92%, and 29 strains had 88% similarity to CAS1. A further 124 (13%) isolates were found to be part of the CAS super family of strains.

**Figure 1:**
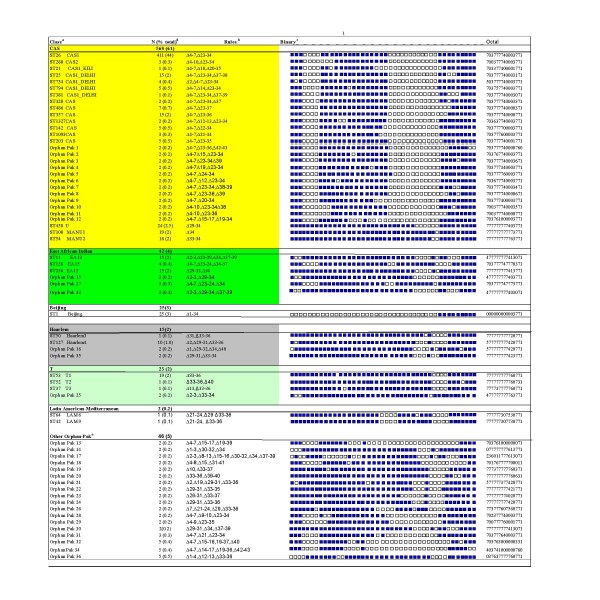
**Spoligotypes shared by Pakistani *M. tuberculosis *isolates**. ^a ^Genotypes as identified in SpolDB4.0 including related Orphan-Pak clusters. ^b ^Rules as defined by absence of spacers (3). ^c ^Filled boxes represent positive hybridization while empty boxes represent absence of spacers. ^d ^Number of isolates. ^e ^Orphan Pak clusters not identified as genotype of any phylogenetic lineage.

The shared types identified in this study are presented in Fig 1 in order of descending cluster size. We have previously identified cluster groups Pak 1–10 in our population [32]. An additional 26 clusters, Pak 11–36 not defined within SpolDB4.0 are described here. Orphan Spoligotypes Pak 1–12 displayed 92–98% similarity with CAS1 and its sub-families and were classified as CAS genotype strains. Manu strains characterized by absence of spacer 33–34 have been included in CAS genogroup [10]. These are presumed to be the probable ancestor of CAS strain types. Pak15, Pak27 and Pak33 showed 88% similarity to EAI strains and were classified with East African-Indian genotype. Whereas, Pak16 and Pak35 had 82% similarity to Haarlem genotype strains.

**Figure 2:**
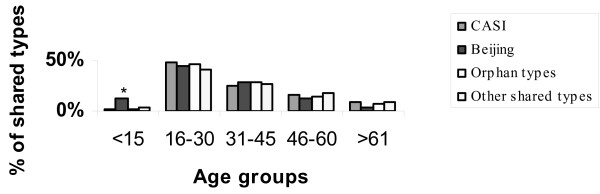
**Distribution of *M. tuberculosis *shared types by age**. The graph illustrates number of clustered spoligotypes isolated from the different age groups expressed as a percentage of total isolates in that cluster (% of clusters). * Beijing strains showed significant association with < 15 year age group (P = value 0.03 Pearson's Chi Square test).

An overall analysis of strain distribution showed that 3/25 (12%) of Beijing strains were isolated from the under 15 years age group as opposed to 8/544 (1.5%) of CAS strains, 3/205 (1.5%) orphan strains and 12/152 (7.9%) of the other shared types (Fig 2). Our data suggests a significant association of Beijing strains with the younger age group (P value = 0.03, Chi square test).

**Figure 3:**
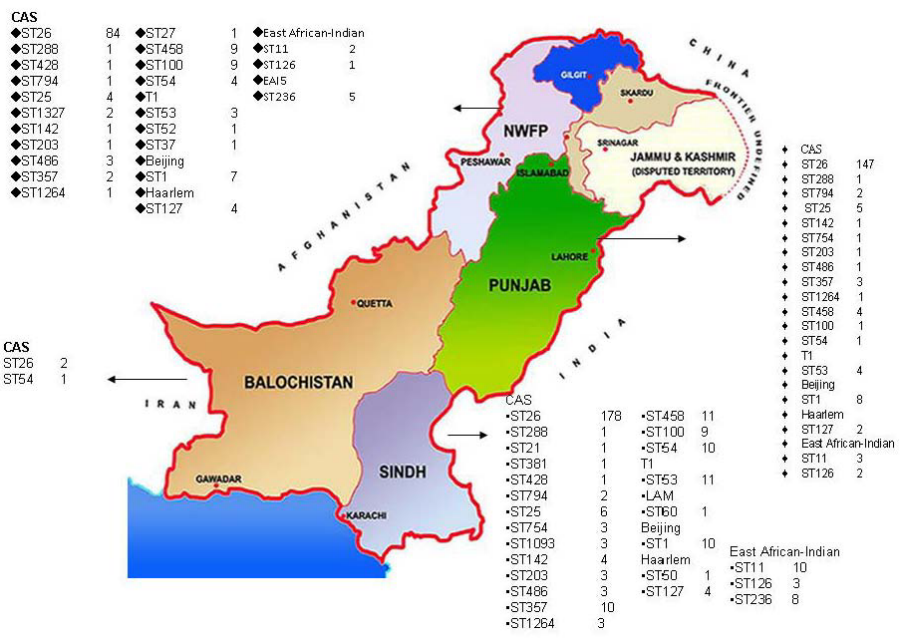
**Geographical distribution of *M. tuberculosis *shared types in Pakistan**. The map describes the geographical distribution of different genotypes in the Sindh, Punjab, NWFP and Baluchistan provinces. The prevalence of CAS genotype and predominant clusters in each province is depicted. Clades: Beijing ;Central Asian strains CAS; Central Asian sub-families; T1 ill-defined T family; Haarlem H strains; East African Indian strain EAI strains.

*M. tuberculosis *genotypes distribution from the four provinces of the country is illustrated in (Fig 3). The largest number of strains was from Sindh followed by Punjab, NWFP and Balochistan. Central Asian strain 1 (CAS1), CAS sub-families (CAS_DEHLI, CAS, U, MANU1 and MANU2) and Beijing strains were prevalent across the country, while EAI strains, T1 family, LAM and Haarlem strains were also present in the different provinces. A comparison of spoligotype distributions between the four regions indicated that the occurrence of CAS1 clade was significantly higher in the Punjab (P < 0.01, Pearsons Chi-square test) as compared with Sindh, NWFP and Balochistan.

### Deletion analysis

The TbD1 region has been identified as an evolutionary marker in *M. tuberculosis *strain lineages [41]. Total 36 *M. tuberculosis *strains were tested for the TbD1 region, 2 strains each from CAS1 and its sub-families (ST25, ST428, ST754, ST794, ST1327, ST288, ST142, ST1093, ST203, ST100, ST54, ST458, and ST21) and in Beijing, Haarlem, EAI3 and EAI5 genotypes. TbD1 region was found to be deleted in all of the above genotypes studied except for the EAI lineages, confirming that the predominant CAS1 strains in this population belongs to a "modern" TB lineage while that EAI strains harboring the TbD1 region and are likely to be "ancestral strains"[43].

### Pulmonary versus extra pulmonary isolates

We next determined genotypes of *M. tuberculosis *strains as identified from either pulmonary [n = 850; Sputum (n = 722) and Bronchial lavage (n = 128)] and extra pulmonary sources [n = 76; Pleural fluid (n = 30), Pus (n = 26), Urine (n = 8) and Tissue (n = 12)]. Table 1 lists these in sequence of genotypes given in Fig 1.

**Table 1:** *M. tuberculosis *shared typed distribution amongst pulmonary and extra-pulmonary disease isolates.

Spoligotype *	**Pulmonary n(%)**	**Extra-pulmonary n(%)**
**CAS**	531(62)	37(49)
**East African-Indian**	37(4)	5(6)
**Beijing**	24(3)	1(1)
**Haarlem**	15(2)	0
**T**	20(2)	3(4)
**LAM**	2(0.2)	0
**Other Orphan-Pak types**^**a**^	41(5)	5(6)
**Orphan types**^**b**^	180(21)	25(33)**

**Total**	**850**	**76**

* Strain genotypes as listed in Fig 1.

^a^Include clustered Orphan-Pak types (Pak 13–14, 17–24, 26, 28–32, 34 and 36)

^b^Include unclustered Orphan types

**Denotes significant difference between pulmonary and extra-pulmonary sources, (p < 0.05, Chi x^2 ^test)

There was no statistically significant difference in the distribution of shared spoligotypes i.e in CAS1, Beijing, EAI, Haarlem, T, LAM and other Pak clusters between their pulmonary and extra pulmonary sources. However, 33% of the isolates from extra pulmonary sources were orphan types, compared with 21% of the pulmonary isolates and this difference was statistically significant (Pearsons Chi-square test P = 0.018; 95% CI, 2.0 to 23.5).

### Drug resistance patterns

We determined the susceptibility pattern of strains to investigate a relationship between *M. tuberculosis *strain prevalence and drug resistance. Drug resistance patterns of Beijing, LAM, T1, EAI, Haarlem, Orphan Pak, and Orphan types were compared with resistance patterns of CAS genotype (see Table 2 panel-1 In-Bold). Resistance to Streptomycin, Rifampicin, Ethambutol, Isoniazid, Pyrazinamide was determined in CAS genotype including sub-groups of CAS family and related Pak-clusters. Of the 926 isolates included in this study, 405 (44%) were sensitive to all 5 first line agents tested and 402 (43%) were MDR.

**Table 2:** Drug resistance patterns of predominant *M. tuberculosis *shared types as compared with genotype of CAS lineage.

clusters**	**Sensitive** **n(%)**^**a**^	**Drug resistant isolates (n) as a percentage of total isolates in the clades (%)**					
		**Rifampicin**	**Isoniazid**	**Streptomycin**	**Ethambutol**	**Pyrazinamide**	**Total**

**CAS**	**245 (45)**	**252 (46)**	**258 (47)**	**141 (26)**	**210 (39)**	**297 (55)**	**568**

							

**EAI**	17 (35)	30 (61)	32 (65)	17 (35)	24 (49)	29 (59)	42
P value		0.08	0.06	0.12	0.13	0.28	
Odds ratio		1.71	1.78	1.73	1.64	1.4	
95% CI		0.92–3.19	0.96–3.30	0.86–3.51	0.86–3.14	0.75–2.62	

**Beijing**	8 (32)	18 (72)	17 (68)	12 (48)	15 (60)	16 (64)	25
P value		0.071	0.1	0.041*	0.08	0.25	
Odds ratio		2.18	2.01	2.6	2.18	1.65	
95% CI		0.93–5.12	0.85–4.76	1.04–6.52	0.90–5.26	0.69–3.92	

**Haarlem**	9 (60)	4 (27)	6 (40)	Not resistant	4 (27)	5 (33)	15
P value		0.16	0.25		0.67	0.16	
Odds ratio		0.43	0.52		0.772	0.45	
95% CI		0.13–1.42	0.17–1.59		0.23–2.55	0.15–1.38	

**T**	6 (30)	8 (40)	9 (45)	5 (25)	5 (25)	7 (35)	23
P value		0.63	0.5	0.54	0.96	0.94	
Odds ratio		1.29	1.42	1.44	0.97	0.96	
95% CI		0.44–3.79	0.5–4.0	0.43–4.8	0.29–3.23	0.31–3.90	

**LAM**	1 (50)	1 (50)	1 (50)	1 (50)	1 (50)	1 (50)	2
P value		0.98	0.97	0.69	0.91	0.89	
Odds ratio		0.97	0.95	1.73	1.61	0.82	
95% CI		0.06–15.03	0.05–15.26	0.10–27.99	0.07–18.76	0.05–13.25	

**Other Orphan-Pak**^**b**^	20 (38)	19 (36)	21 (40)	9 (17)	19 (36)	13 (24)	46
P value		0.93	0.99	0.55	0.75	0.08	
Odds ratio		0,97	0.99	0.78	1.1	0.53	
95% CI		0.51–1.85	0.52–1.88	0.34–1.76	0.57–2.13	0.26–1.1	

**Orphan types**	96 (47)	106 (52)	137 (67)	130 (63)	177 (86)	129 (63)	205
P value		0.67	0.058	0.001*	0.001*	0.52	
Odds ratio		1.03	1.35	2.35	2.15	1.1	
95% CI		0.77–1.48	0.99–1.85	1.68–3.29	1.57–2.93	0.81–1.1	

^a^Sensitive to first line drugs (Rifampicin, Isoniazid, Streptomycin, Ethambutol and Pyrazinamide)

^b^Includes a total of 18 clusters

**Strain genotypes as listed in Fig. 1

* Significant difference compared to CAS genotypes

The association between *M. tuberculosis *genotypes and first line drug resistance was assessed by logistic model in comparison with CAS genotype, a significantly higher resistance was observed amongst 'Orphan' isolates and to streptomycin (P value = 0.001, OR; 2.35, CI; 1.68–3.29) and ethambutol (P value = 0.001, OR; 2.15, CI; 1.57–2.93) while, Beijing strains showed higher resistance to streptomycin (P value = 0.041, OR; 2.6; CI; 1.04–6.52).

Analysis of cluster types further showed that Beijing strains (P value = 0.01, Pearsons Chi-square test), and EAI (P value = 0.001, Pearsons Chi-square test) were associated with MDR. The MDR rate in the predominant CAS genotype was not found to be statistically significant (P value = 0.36, Pearsons Chi-square test).

## Discussion

This study presents novel information regarding genotypic diversity and drug resistance of *M. tuberculosis *strains in Pakistan. We found that 411 (44%) of 926 study isolates to be CAS1 or ST26 strains [25], confirming previous reports of 39% prevalence of CAS1 amongst *M. tuberculosis *strains [32]. The results further showed that in addition to ST26 the majority of spoligotypes belonged to the CAS genotype. CAS1 has been also identified by recent studies as a predominant strain in Delhi[25] and Mumbai[44]. Prevalence of Beijing strains in our study at 3% (n = 25) compares well with data from Delhi, where 8% of 105 isolates are reported to be of Beijing family[25]. We further identified 36 clusters (Pak clusters) not identical with any of the STs described within SpolDB4.0.

Comparison of pulmonary and extra pulmonary sources showed a significant association of 'Orphan' spoligotypes with extra pulmonary disease. This difference between pulmonary and extrapulmonary spoligotypes may be attributed to the greater ease with which pulmonary strains may be transmitted via aerosol routes, leading to greater transmission and strain clustering.

The global population structure of *M. tuberculosis *is reportedly defined by six phylogeographical lineages, each associated with specific human populations [29,45]. It has been suggested that particular lineages of *M. tuberculosis *might be adapted to specific human populations and maladapted to others[45]. Strain differences in different geographical regions may be linked to different ethnic subpopulations in these regions, and their migration histories [46].

The CAS strains have been shown to be predominant [30,47] in this region, the distribution of CAS1 strains was greater in the province of Punjab (P < 0.01, Pearsons Chi-square test) as compared with Sindh, NWFP, and Balochistan. This could be due to the shared border and similar population mix between Punjab now in Pakistan (West Punjab prior to the 1947 partition of India), and East Punjab, (now Punjab, North India).

Although not statistically significant, East African-Indian strains were more prevalent in Sindh. The fact that 4% of our isolates were East African-Indian is comparable to an earlier study from Delhi, reporting 8% EAI strains in their population [25]. Both these figures are in contrast to a study from Southern India indicating an 80% prevalence of TbD1+/EAI isolates amongst their samples [43]. It has been hypothesized that lineages that are rare in specific human population are not adapted to spreading within these populations and show a significantly lower case-rate ratio. This hypothesis is supported by a recent report[30] suggesting that TB in India is essentially caused by historic clones of tubercle bacilli which circulate with geographic predilection.

Deletion analysis demonstrated the absence of TbD1 region in CAS1 and its sub-families. A recent study [43] also suggests that CAS1 family evolved as a result of an evolutionary event causing TbD1 deletion from a common ancestor[18,41]. The study further suggests that strains within CAS genotype are related through minor genetic changes. The similarity seen in our study between Pak clusters 1–12 and CAS genotype suggests an evolution from a common ancestor belonging to the same phylogenetic lineage CAS.

Haarlem4 and T1 were also identified amongst our isolates. It is reported that more than 60% of ST127 (H4, modern type) are localized in Iran and Russia. Their presence in our population suggests the role of traditional migratory routes from central Asia in the history of tuberculosis. In addition to the predominant groups, we also found clusters of rare/localized shared types listed in SpolDB4.0 that have previously been found only in North America, Australia and Europe (CAS1_KILI) as well as those found in neighboring Iran and India (LAM 9, LAM 6).

In the absence of national surveillance for drug resistance in Pakistan, the majority of published reports rely upon laboratory data based on passive specimen collection. There is thus little information about the burden of MDR at a community level. WHO estimates suggest an MDR rate of under 3% amongst new cases and 20–40% amongst previously treated TB cases[48]. A recent report of 1.8% MDR-TB, in untreated cases in Pakistan corroborates the WHO estimates [49]. Since the samples in our study were not collected through active case finding, the high MDR rate noted in our specimens is likely to reflect the large number of previously treated patients included in this study. This however, cannot be confirmed due to absence of prior treatment history for our patients.

In agreement with an earlier report [32] CAS1 and related CAS sub-family spoligotypes in this study showed no correlation with MDR-TB. However, a higher relative risk of MDR amongst Beijing strains was noted. Association between Beijing strains and MDR varies worldwide [50,51], A recent study from Mumbai, India reported a higher frequency of Beijing strains (35%) amongst MDR isolates [44]. The distinctive feature of Beijing MDR-TB outbreak is accelerated transmission as compared with other MDR-TB outbreaks[52]. Further, East African-Indian strain was also found to be associated with MDR supported by a study from Iran [53]. Recent studies moreover suggests that MDR-TB strains may be responsible for emergence of XDR-TB cases [53,54].

The higher occurrence of streptomycin resistance in Beijing strains as compared to CAS genotype has importance in that particular lineage of *M*. *tuberculosis *might harbor polymorphisms which make them resistant to certain anti-tuberculosis drugs.

In our study population, association of Beijing strains with a younger age group (<15 years) is concerning. It suggests that Beijing strains may be an emerging strain type and is likely to increasingly contribute to the burden of drug resistant tuberculosis in this region.

## Conclusion

The identification of a dominant spoligotype, CAS, similar to previously identified isolates in India and Bangladesh illustrates an important trend in the *M. tuberculosis *infection pattern in the South Asian region. All predominant clusters apart from CAS1 strains were equally distributed in the country suggest a continual transmission of strains. This data presents a comprehensive evaluation of the strain-to-strain variability in *M. tuberculosis*, important in phenotypic consequences, also these phylogeographical strain variation may affect the development of new diagnostic tools, drugs, and vaccines for treatment in the endemic region.

## Competing interests

The authors declare that they have no competing interests.

## Authors' contributions

MT conducted the experimental work and prepared the manuscript. ZH supervised the laboratory work and study design. AK and AA provided technical help with spoligotyping. RS provided statistical data analysis support. SG provided advice regarding MTB strain typing. RH planned the idea and supervised the study. All authors read and approved the final manuscript.

## Pre-publication history

The pre-publication history for this paper can be accessed here:


